# The consortium project INTEGRATE-ADHD - comparison and integration of administrative and epidemiological ADHD diagnostic data by clinical assessment: study description, non-responder analysis, and sample characteristics

**DOI:** 10.1186/s12889-026-26569-0

**Published:** 2026-02-16

**Authors:** Ann-Kristin Beyer, Lilian Beck, Stefan Pfeifer, Ronny Kuhnert, Heike Hölling, Thomas Jans, Annalena Berner, Leila Hetzke, Sophia Weyrich, Vanessa Scholz, Theresa Emser, Diana Mager, Sanna Ulsamer, Chantal Wallau, Marcel Romanos, Martha Gilbert, Anne Kaman, Ulrike Ravens-Sieberer, Julian Witte, Lena Hasemann, Katharina Weinert, Wolfgang Greiner, Jonas Widmann, Anna Grau, Anna Schäfer, Cornelia Fiessler, Peter Heuschmann, Cordula Riederer, Robert Schlack

**Affiliations:** 1 Department of Epidemiology and Health Monitoring, Robert Koch Institute, Gerichtstraße 27, 13347 Berlin, Germany; 2 Centre of Mental Health, Department of Child and Adolescent Psychiatry, Psychosomatics and Psychotherapy, University Hospital Würzburg, Würzburg, Germany; 3 Donders Institute for Brain, Cognition and Behaviour, Radboud University, Nijmegen, The Netherlands; 4 Department of Child and Adolescent Psychiatry, Psychotherapy, and Psychosomatics, Research Section Child Public Health, University Medical Centre Hamburg-Eppendorf, Hamburg, Germany; 5 Vandage GmbH, Bielefeld, Germany; 6 Department of Health Economics and Health Care Management, Bielefeld University, Bielefeld, Germany; 7 Institute of Clinical Epidemiology and Biometry, Julius Maximilian University of Würzburg, Würzburg, Germany; 8 Clinical Trial Centre, University Hospital Würzburg, Würzburg, Germany; 9 Institute for Medical Data Sciences, University Hospital Würzburg, Würzburg, Germany; 10 DAK-Gesundheit, Hamburg, Germany

**Keywords:** ADHD, Administrative, Epidemiological, Prevalence, Clinical assessment, Data-linkage

## Abstract

**Background:**

This paper aims to describe the study design, methodological approach, conduct, and sample characteristics of the data linkage project INTEGRATE-ADHD. The project was designed to evaluate the concordance and validity of administrative versus epidemiological and clinical ADHD diagnoses, thereby providing insights for health care and health care planning. The assessment of treatment satisfaction among families with children with ADHD, as well as the health economics of ADHD, is also part of the project.

**Methods:**

A total of 24,880 parents of children and adolescents statutorily insured with DAK-Gesundheit, who had at least one confirmed administrative ADHD diagnosis in one quarter of the 2020 insurance year, were invited to complete an online survey. The survey included questions on ADHD diagnosis, disorder-specific and comorbid psychopathology, health care utilisation, and both the quality of and satisfaction with health care. A random sampling procedure was applied to select 202 participants for a guideline-based clinical online assessment. Administrative, survey, and clinical diagnostic data were subsequently linked at the individual level. Non-responder analyses and sample characteristics were examined with descriptive statistics. Group differences were tested with chi-square and t-tests. Sample representativeness was evaluated.

**Results:**

A total of 5,461 parents of youths (mean age = 12.5 years; 25.4% girls) participated in the survey (response rate: 21.5%). A guideline-based clinical ADHD decision could be made for a subsample of 201 participants (mean age = 12.1 years; 27.9% girls). Non-responder analyses indicated only minor differences in sociodemographic and health care–related variables, parents of more severely affected children and adolescents were more likely to participate in the survey. Weighting factors were calculated to adjust for these deviations.

**Conclusion:**

Non-responder analyses and comparisons with nationwide outpatient diagnostic data suggest that the results are broadly generalisable to German children and adolescents with an administrative ADHD diagnosis. By linking administrative, epidemiological, and clinical ADHD diagnostic data at the individual level, INTEGRATE-ADHD facilitates an individual-level evaluation of how administrative ADHD diagnoses relate to epidemiological and clinical assessments.

## Background

Attention-deficit/hyperactivity disorder (ADHD) is one of the most commonly diagnosed mental health disorders among children and adolescents, both in Germany and globally, with prevalence rates around 5% [1–3]. Population-based prevalence rates are typically derived from claims (administrative) data or epidemiological surveys and constitute a crucial basis for health care planning. Survey data and administrative data, however, are limited in their comparability and are sometimes even contradictory, for instance regarding the magnitude of prevalence or temporal prevalence trends.

Getahun and colleagues, for example, reported a prevalence of 4.9% for ADHD diagnoses among 5- to 11-year-olds in Southern California over a ten-year period (2001–2010), based on U.S. health insurance data [4]. In contrast, data from the 2007 National Survey of Children’s Health (NSCH), relying on parental reports of a health care provider’s diagnosis, indicated a national ADHD prevalence of 9.5% among children aged 4–17 years [5]. After adjusting the NSCH sample to match the characteristics of Getahun’s study population, Visser and colleagues [5] estimated a prevalence of 4.7% for 5- to 11-year-olds in California, which closely approximated the 4.9% reported by Getahun et al. [4]. The authors concluded that these findings supported the convergent validity of parent-reported ADHD diagnoses, although without linking data at the individual level [5].

In Germany, ADHD prevalence estimates among children and adolescents based on health insurance data increased sharply at the beginning of the 21st century, whereas large population-based epidemiological studies—such as the German Health Interview and Examination Survey for Children and Adolescents (KiGGS), which relies on parent-reported lifetime diagnoses—reported stable or declining rates [3, 6, 7]. For example, between 2006 and 2014, ADHD prevalence in data from Barmer, the second-largest statutory health insurer in Germany, increased by 66% (from 2.9% to 4.2%) among individuals aged 0–19 years [8]. In contrast, KiGGS data based on parental diagnostic reports indicated a 17% decrease (from 5.3% to 4.4%) between the survey periods 2003–2006 and 2014–2017 [3]. The clinical validity of both data sources remains unknown.

Discrepancies may arise due to differences in data sources, measurement methods, or population characteristics, with varying definitions, differing inclusion and exclusion criteria, and different reference periods (e.g., annual prevalence in claims data vs. lifetime prevalence in survey data) being among the key contributing factors [9]. Health insurance data are primarily collected for billing purposes and reflect health care utilisation whenever a service is provided. These data typically reflect the insured population of a specific insurer. In contrast, surveys aim to be population representative. Hospitalised patients are generally excluded from surveys, which could result in biased morbidity estimates. In addition, surveys often lack clinical diagnostics, particularly for conditions that involve complex diagnostic processes, such as ADHD. Instead, they rely on self-reported diagnoses from participants, which are typically presented as lifetime, 12-month, or point prevalence. The increase in ADHD diagnosis rates among youth has also been attributed to overdiagnosis, with ADHD sometimes seen as a ‘trendy’ diagnosis [10].

To investigate the potential causes of the aforementioned discrepancies, the consortium project INTEGRATE-ADHD was launched. The project’s main objective is to integrate and compare administrative and epidemiological diagnostic data as well as clinical assessments based on established guidelines at the individual level. To this end, parents of children with an administrative ADHD diagnosis were surveyed online using modified epidemiological questionnaires from the KiGGS study [11–13] and its in-depth module on child mental health (BELLA study; [14]). The questionnaires included, among other topics, questions about the child’s ADHD diagnosis. A subsample of youths was also clinically assessed according to national ADHD diagnostic guidelines [15]. The administrative, survey, and clinical data were subsequently linked at the individual level to create an integrated dataset.

The following objectives are addressed by INTEGRATE-ADHD:

- To evaluate the extent to which administrative ADHD diagnoses in children and adolescents are reported by their parents.
- To assess the degree to which administrative ADHD diagnoses in children and adolescents are clinically confirmed using guideline-based ADHD diagnostics.
- To examine treatment satisfaction and health care service utilisation.
- To analyse the direct costs associated with an administrative ADHD diagnosis in children and adolescents.

This paper provides an overview of the study design and conduct of the INTEGRATE-ADHD project and presents, additionally, the results of the non-responder analysis as well as the basic sample characteristics.

## Design and methods

### Inclusion criteria and case definitions

The consortium project INTEGRATE-ADHD was carried out as a cross-sectional interview and examination survey of parents (respondents) of youths (cases) insured with DAK-Gesundheit, the third-largest German statutory health insurance company. Children and adolescents were included if they had at least one confirmed outpatient or inpatient ADHD diagnosis (ICD-10 F90.0–9, main and/or secondary diagnosis) in 2020 (M1Q criterion), were born between 2003 and 2020, and had not changed their health insurance provider during 2020.

### Sampling and conduct of the online survey

**Fig. 1:**
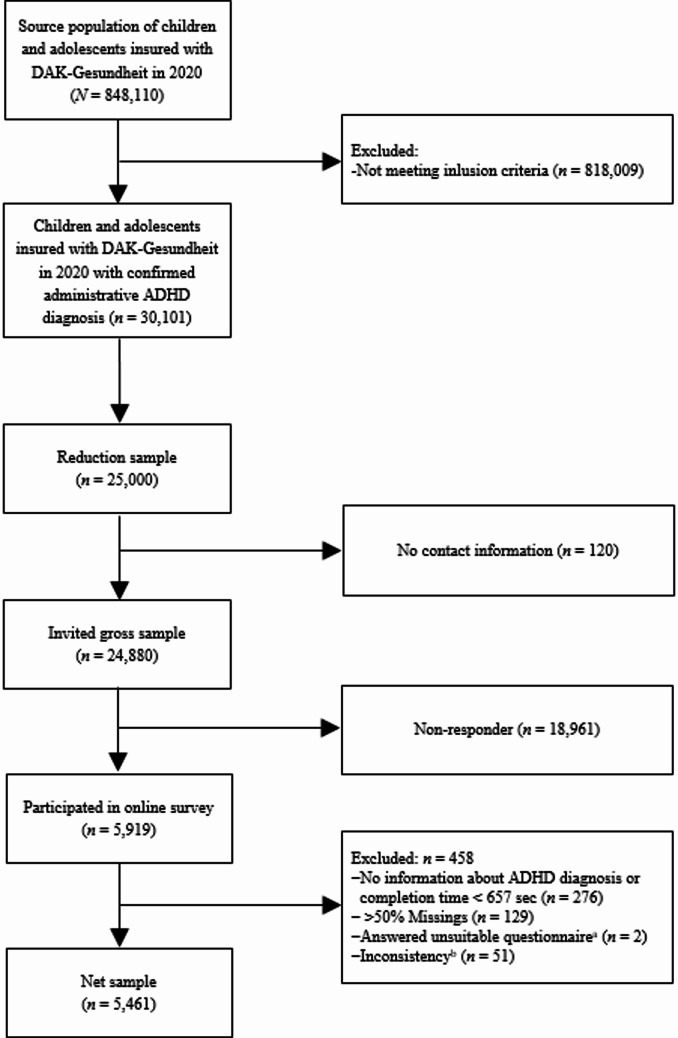
Flow chart of sampling and study participation. ^a^due to incorrect entry of the child’s age. ^b^inconsistent information on age or gender in the administrative and the online survey data after data-linkage

The source population of the study comprised 848,110 children and adolescents insured with DAK-Gesundheit in 2020 (see Fig. 1). Originally, a full survey of the parents of all children and adolescents insured with DAK-Gesundheit who met the inclusion criteria (approximately 25,000) was planned for the online survey. The resources were calculated and requested accordingly. However, between the study application and the approval of the study, the number of children and adolescents insured with DAK-Gesundheit had increased by approximately 200,000 due to the takeover of another health insurance company. Since the funding could not be adjusted, a probabilistic reduction sample was drawn from the actual 30,101 children and adolescents who met the inclusion criteria in order to return to the funded number of 25,000 children and adolescents. Of these, 120 families could not be contacted due to missing contact information or refusal to be contacted by DAK-Gesundheit, resulting in a final sample of 24,880 parents or legal guardians (hereafter referred to as parents) who were contacted and invited to participate in the study.

The online survey was conducted in four waves between October 2021 and August 2022 (first wave: October 2021, second wave: January 2022, third wave: April 2022, fourth wave: June 2022). Parents of the selected children received a postal invitation from DAK-Gesundheit, which included brief information about the study and data protection, as well as a link to access the questionnaire and a personal identification number (PIN). Upon following the link, parents received comprehensive information about the study’s objectives and procedures, the linkage of their survey and administrative data, and data privacy. Before starting the questionnaire, parents gave their informed consent by clicking a confirmation box. Similarly, youths aged 14 years and older provided their informed consent separately for their parents to share information about them. Upon completion of the questionnaire, participants received a €30 shopping voucher as an incentive.

### Sampling and conduct of the clinical assessment

After completing the online questionnaire, parents were asked if they would be willing to participate in an additional clinical assessment with their child. Children aged 14 years and older were asked for their consent separately. In total, 3,511 families (i.e., 59.3% of all online survey participants) agreed.

Of these, a subsample was selected for the clinical assessment using a stratified random procedure. The planned analyses included a comparison of two equally sized groups of children and adolescents with (ED+) and without (ED-) an epidemiologically parent-reported ADHD diagnosis. With a sample size of *n* = 100 each for ED+ and ED- groups, the assumed proportion of clinically confirmed ADHD diagnoses of 95% in the ED+ group was estimated with a 97.5% confidence interval of [0.88; 0.98], while the 97.5% confidence interval for the assumed proportion of 50% clinically confirmed ADHD diagnoses in the ED- group was [0.39; 0.61]. Because the ED- group was expected to be less frequent than the ED+ group, an oversampling of the ED- group was conducted, resulting in a preselected pool of 576 subjects (288 ED+ and 288 ED-) for the clinical assessment. Surplus sampling was employed in anticipation that some participants would withdraw their consent. There were no significant differences in demographics, sociodemographics, or health care-related characteristics between individuals who had consented to participate in the clinical assessment and were preselected but did not participate for various reasons, and those who consented, were selected, and participated—except regarding individuals who already had an ADHD diagnosis in the previous year and those whose initial diagnosis was made by a paediatrician: the proportion of the former was significantly higher among participants of the clinical assessment, whereas the proportion of the latter was significantly lower.

**Fig. 2:**
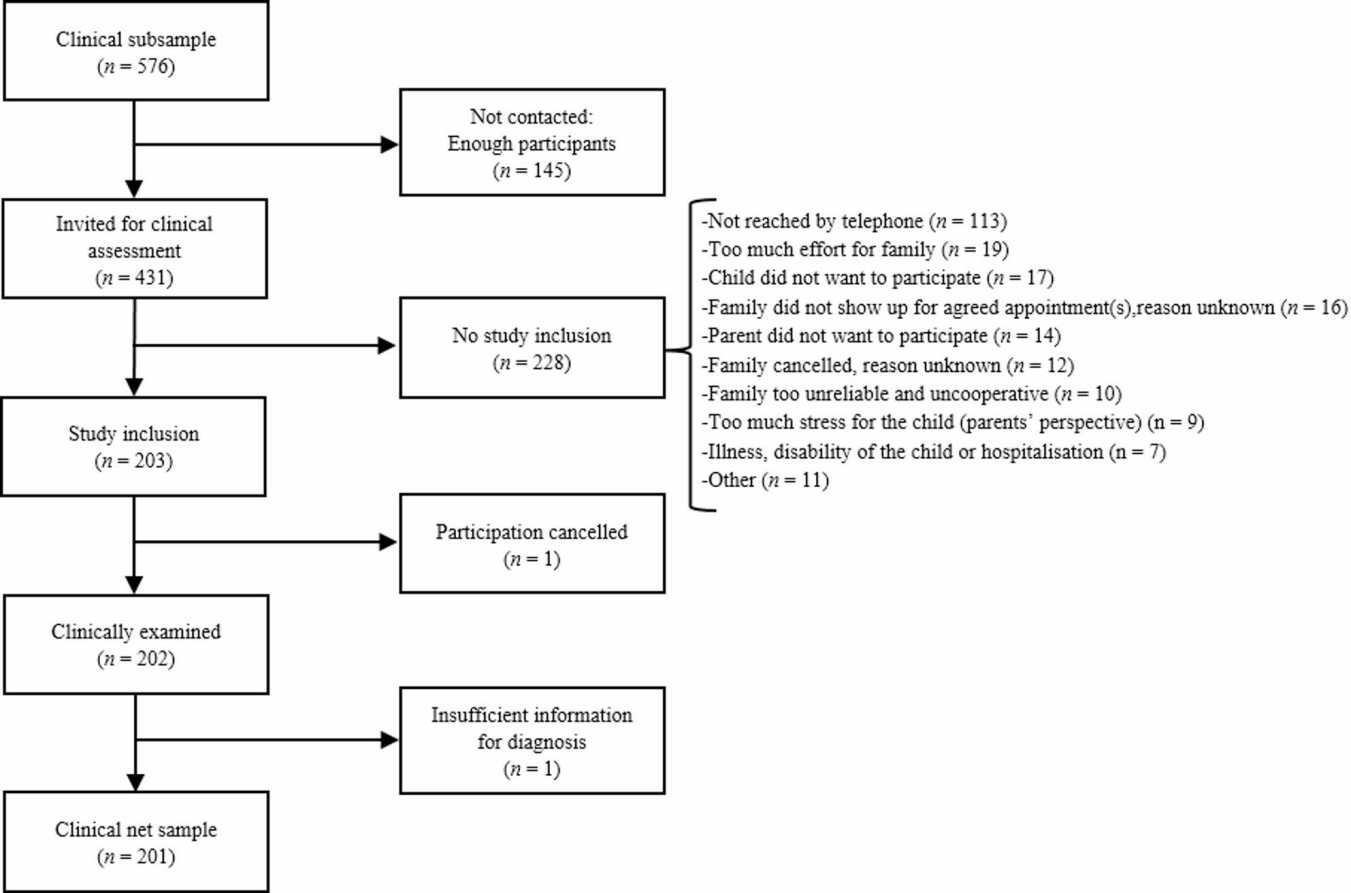
Flow chart of clinical subsampling and participation in clinical assessment

The clinical assessment took place between January 2022 and January 2023. It was conducted online by psychologists and psychotherapists in training, following the current German guideline ‘Attention Deficit/Hyperactivity Disorder (ADHD) in Childhood, Adolescence, and Adulthood’ [15]. The preselected families first received an invitation letter, followed by a telephone call providing general information about the aims and procedures. Subsequently, 431 families received a letter containing more detailed information, a consent form, and a confidentiality release form. Ultimately, a total of 203 families participated in the clinical assessment (see Fig. 2). After completion, the families received a written report of the results and a €200 incentive.

### Topics and instruments of the online survey

In the online survey, questionnaires from the German Health Interview and Examination Survey for Children and Adolescents (KiGGS study; [11–13, 16]) and its in-depth module on child mental health (BELLA study; [14, 17]) were used. Age-specific, self-administered, parent-rated questionnaires were employed for the following age groups: 0–2, 3–6, 7–10, 11–13, and 14–19 years (based on the child’s age at the time of the survey). These included proven items and validated instruments related to ADHD (e.g. the German Fremdbeurteilungsbogen FBB-ADHS, the restless-impulsive subscale of Global Index Connor’s Scale, or the Parental ADHD symptom screening), psychopathology (e.g. the Centre for Epidemiological Studies Depression Scale for Children, the Screen for Child Anxiety-Related Emotional Disorders or the Externalising subscale of the Child Behavior Checklist), risks and resources, as well as health-related quality of life (see Table 1). Socio-demographics, child height and weight, risk and protective factors such as premature birth, postnatal complications, birth size and weight, maternal smoking and alcohol consumption during pregnancy, current parental smoking, the child’s exposure to smoke, and health care utilisation were also assessed [11, 14, 16].

**Table 1:** Validated instruments in the INTEGRATE-ADHD online survey

Construct	Age group	Instrument/Method
**Child and parental ADHD symptoms**		
ADHD symptom screening	0–6	Fremdbeurteilungsbogen für Aufmerksamkeitsdefizit-/Hyperaktivitätsstörungen (FBB-ADHS-V; [18]), preschool version
	5–19	FBB-ADHS ([18])
ADHD symptom screening	5–19	Restless-impulsive subscale of Global Index Connor’s scale ([19, 20])
ADHD symptom screening	3–19	Hyperactivity/inattention subscale of Strengths and Difficulties Questionnaire (SDQ; [21])
Parental ADHD symptom screening	parents	ADHD Self-Report Scale (ASRS; [22–24])
**Other child and parental psychopathological symptoms**		
Emotional problems and peer problems	3–19	Emotional problems and peer problems subscales of SDQ ([21])
Impact of mental health problems	3–19	SDQ impact supplement ([25])
Depressive symptoms	7–19	Centre for Epidemiological Studies Depression Scale for Children (CES-DC; [26])
Symptoms of anxiety	7–19	Screen for Child Anxiety-Related Emotional Disorders (SCARED; [27, 28])
Irritability	7–13	Diagnostic Tool for Affective Dysregulation in Children—Screening Questionnaire (DADYS-Screen; [29])
Irritability	7–19	Patient Reported Outcomes Measurement Information System Anger Scale (PROMIS Anger-Scale; [30, 31])
Disruptive behaviour disorders	0–6	Externalising subscale of the Child Behavior Checklist 1,5–5 years (CBCL 1,5–5; [32])
	7–19	Externalising subscale of the Child Behavior Checklist (CBCL; [33])
Parental psychopathology	parents	Short version of the Symptom Checklist (SCL-K-9; [34])
Parental strain	parents	Parental Strain Scale ([35])
**Risks and resources and health-related quality of life**		
Personal resources	7–19	Personal Resource Scale ([36])
Social support	7–19	Adapted version of the Social Support Scale ([37])
Family cohesion	0–19	Family Climate Scale ([38])
Parents’ health literacy	parents	16-item version of the European Health Literacy Survey ([39])
Health-related quality of life	7–19	KIDSCREEN 27 ([40])

With regard to ADHD, parents were asked whether their child had ever been diagnosed with ADHD by a medical doctor or psychologist, and whether the disorder had been present in the last 12 months [6, 7, 41, 42]. If parents reported an ADHD diagnosis for their child, they were asked whether they accepted the diagnosis, when it was first made, whether the child had ever received ADHD medication, and if so, which medications had been used in the past seven days. They were also asked whether they had utilised further treatment, whether they sought treatment in 2020 due to their child’s ADHD, and whether they perceived their child as being discriminated against in kindergarten or at school. Parental ADHD was assessed for both mother and father by asking respondents about a lifetime diagnosis by a physician or psychologist. If a parental diagnosis was reported, respondents were asked when the diagnosis was made.

Furthermore, respondents were asked about the child’s utilisation of paediatric, psychiatric, psychotherapeutic, or general medical health care, as well as alternative practitioners, physiotherapists, occupational therapists, or speech therapists in the past 12 months. If any of these services had been used, respondents were asked about their satisfaction with the treatment. If no utilisation was reported, respondents were asked about potential barriers.

To address potential issues related to the COVID-19 pandemic, the questionnaire included additional items concerning the child’s school and family situation, peer contact, and the general living conditions during the pandemic [43].

### Topics and instruments of the online clinical assessment

The clinical assessment, conducted in accordance with the S3 guideline of the Association of the Scientific Medical Societies in Germany [15], comprised two video chat sessions per participant, each lasting between two and four hours. In addition, parents, teachers, and, from the age of 11, the child him- or herself completed online questionnaires. Furthermore, performance tests were conducted online with the child.

The parent was interviewed about basic demographic and anamnestic data and reported the child’s medical history, focusing on both somatic and psychological health as well as previous medication. Psychological stress was evaluated using a screener based on Axis V — abnormal psychosocial conditions — of the multiaxial classification system of ICD-10, chapter V (F) (MAS; [44]). To screen for comorbidity, the “Interviewleitfaden Screen” (ILF-Screen; [45]) was applied to one parent and the child (from eight years of age). In cases of abnormal results, the disorder-specific sections of the Diagnostic Interview for Psychiatric Disorders in Children and Adolescents (Kinder-DIPS; [46, 47]) were administered. Pervasiveness and onset of ADHD symptoms and impairment were assessed using the semi-structured “Interviewleitfaden für Externale Störungen” (ILF-EXTERNAL; [45]). Furthermore, ADHD rating scales from the DISYPS-III were completed by children aged 11 years and older (SBB-ADHS), by the parent (FBB-ADHS / FBB-ADHS-V), and by a teacher or educator (FBB-ADHS / FBB-ADHS-V; [18]). The child’s intelligence was assessed using the online version of Raven’s 2 [48]. To evaluate executive functions, the child completed an online version of the Continuous Performance Test (CPT; [49]). Throughout the entire assessment, the diagnostician rated the child’s behaviour using an observation scale from the DISYPS-III (“Merkmalsausprägungen in der Untersuchungssituation”, [18]). The severity of all psychological symptoms was evaluated with the severity scale of the Clinical Global Impressions Scale (CGI-S; [50]). The assessment also included a review of school reports and previous medical reports on symptoms of inattention, hyperactivity, and impulsivity provided by the families and their treatment providers. Where possible, pretreatment information was also obtained from treatment providers (e.g., physicians or psychotherapists) via telephone.

Based on all the information gathered, the diagnostician made a best-estimate judgment regarding the presence or absence of ADHD, according to ICD-10 and DSM-5 criteria. Each assessment was supervised by both a psychological and a medical expert. For detailed information on the diagnostic procedure, see Hetzke et al. [51].

### Topics and data processing of the administrative data

Administrative data for 2019 and 2020 were provided by DAK-Gesundheit. The administrative data for the youths includes individual demographic characteristics (date of birth, age in 2020, sex, federal state), inpatient and outpatient health care utilisation (including procedures and diagnoses according to ICD-10-GM-2022, medication prescriptions, medical aids, non-physician specialist care, and rehabilitation services), as well as information on direct utilisation costs. Data cleaning involved verifying quantities and cost data for any unreasonable values. Cases with unreasonable data were excluded. Cost values of zero were not excluded, as these could relate to correction invoices or similar situations. Otherwise, the data were reported without further modification.

Some external variables were added to the administrative data set, including a comorbidity index developed by Sun et al. [52], which summarises 24 conditions. The German Index of Deprivation (GISD; [53]), indicating socioeconomic deprivation at the district level (low, medium, high), was also added, along with variables containing regional ratios of physicians and psychotherapists (paediatricians, child and adolescent psychiatrists, medical and psychological psychotherapists, general practitioners) per 100,000 inhabitants at the district level. These were extracted from the register of the Federal Association of SHI Physicians [54]. The 2019 data was used to determine incident ADHD (i.e., an individual disease-free pre-observation period of at least four quarters).

### Data flow and data linkage

**Fig. 3:**
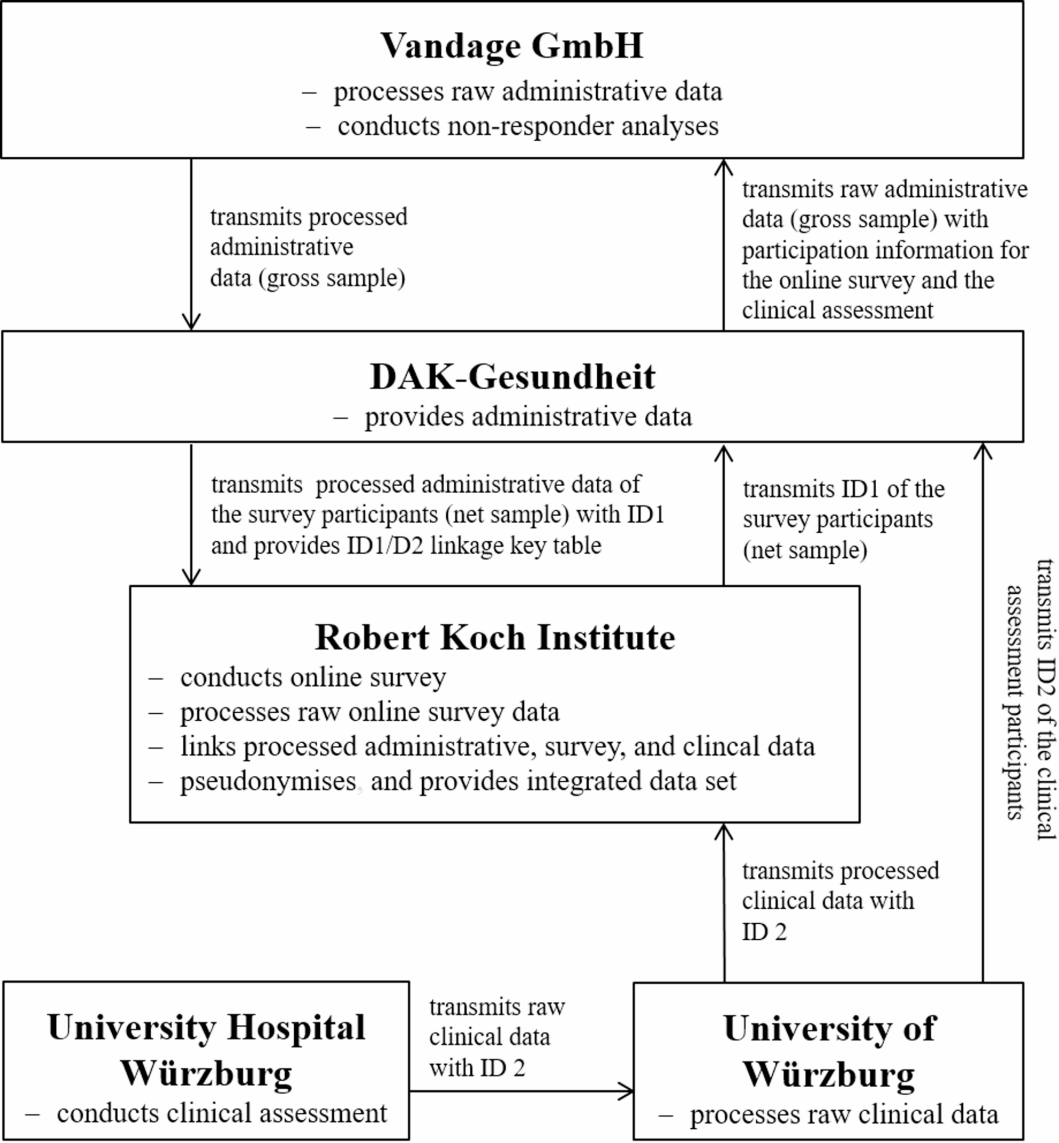
Data flow and data linkage. ID1= identification numbers of the online survey participants; ID2= identification numbers of the clinical assessment participants

Following the completion of data collection, the raw online survey data were processed at the Robert Koch Institute (RKI; see Fig. 3). The raw clinical data were transmitted from University Hospital Würzburg to University of Würzburg and processed at the University of Würzburg. The RKI transmitted the identification numbers of the online survey participants (ID1) to DAK-Gesundheit, while the University of Würzburg transmitted the identification numbers of the clinical assessment participants (ID2) to DAK-Gesundheit. Based on ID1 and ID2, DAK-Gesundheit generated a variable with participation information, added it to the raw administrative data (gross sample), and transmitted this enriched dataset to Vandage GmbH. Vandage GmbH subsequently processed the raw administrative data, conducted the non-responder analyses, and returned the processed administrative dataset to DAK-Gesundheit.

DAK-Gesundheit reduced the processed administrative dataset to those participating in the online survey (net sample), removed the participation information, added ID1, and transmitted the resulting administrative dataset to the RKI. In parallel, the University of Würzburg transmitted the processed clinical data with ID2 to the RKI. DAK-Gesundheit provided the RKI an ID1/ID2 linkage key table, which the RKI used to link the processed administrative, online survey, and clinical data into an integrated dataset. Subsequently, the linked dataset was pseudonymised and made available to the consortium partners for analysis. The data flows and ID-based pseudonymisation were designed to ensure that no consortium partner was able to re-identify individual study participants. The data flow is shown in Fig. 3.

### Data protection and ethical approval

The study was conducted in strict compliance with the data protection regulations of the EU General Data Protection Regulation (GDPR) and the German Federal Data Protection Act (BDSG). The ethical principles for medical research involving human participants, as set out in the World Medical Association’s Declaration of Helsinki, were also observed. The study was reviewed and approved by the Ethics Committee of the University of Würzburg (24 March 2021; reference number 249/20) and registered with the German Clinical Trials Register (DRKS; study number DRKS00028866).

### Statistical analyses

Descriptive analysis regarding response and non-responder analyses were conducted, the sample representativeness of the data was evaluated and weighting factors were calculated. Furthermore, descriptive statistics for sample characteristics of the online sample and the clinical subsample were conducted. Group differences were examined using chi-square tests and t-tests.

## Results

### Representativity

The analyses in this project are based on data from the statutory health insurance fund DAK-Gesundheit, making this a single-insurer study. However, DAK-Gesundheit is the third-largest statutory health insurer operating nationwide in Germany, and, as such, it represents not only a large proportion of children and adolescents with statutory insurance in the country but can also be considered approximately representative of the German child and adolescent population. Comparative analyses conducted as part of the project involving children and adolescents insured with DAK-Gesundheit versus all children and adolescents living in Germany showed only slight deviations between eastern Germany (+1.9%) and western Germany (-1.9%) among children and adolescents insured with DAK.

To assess representativeness with respect to the population with an administrative ADHD diagnosis, available nationwide data on outpatient care (here based on the M2Q criterion) from the Central Research Institute for Ambulatory Health Care in Germany (Zi) for the years 2015 and 2016 [55] were compared with the INTEGRATE-ADHD gross sample. There were only slight deviations in gender distribution in the INTEGRATE-ADHD gross sample (boys: -1.4%, girls: +3.1%); however, younger subjects were overrepresented and older subjects underrepresented (5- to 6-year-olds: +5.3%, 7- to 8-year-olds: +13.4%; 11- to 12-year-olds: -7.9%, 13- to 14-year-olds: -12.5%). In sum, deviations of the gross sample from the total population of children and adolescents in Germany with administrative ADHD diagnosis were small for gender and more pronounced for age, with no possibility for correction.

### Response and non-responder analysis

From a total of *n* = 24,880 invited parents, *n* = 5,919 participated in the online survey (see Fig. 1). Of these, *n* = 458 participants were excluded from the analyses because they either did not answer the question about their child’s ADHD diagnosis, completed the survey in less than 657 s (pure reading time), had more than 50% missing values, or completed an inappropriate questionnaire due to incorrectly entering their child’s age. Consistency checks after data linkage led to the exclusion of additional cases due to inconsistent information on age or gender in the administrative and survey data. This resulted in a net sample of *n* = 5,461. According to AAPOR’s Standard Definitions, Version 9 (RR3), the response rate was 21.5% [56]. This is in line with DAK-Gesundheit’s experiences of a response rate of roundabout 20% in previous surveys with insured persons, when surveying a group for whom the survey topic is of high personal relevance, in particular since lower adherence was to be expected in a survey population with ADHD due to the high heritability of the disorder between parents and their children. In total, *n* = 202 families underwent clinical assessment. Based on the available information, ADHD according to ICD-10 or DSM-5 criteria could not be determined for one child. Thus, clinical ADHD information is available for *n* = 201 children (see Fig. 2).

For the non-responder analysis, the characteristics of responders and non-responders of both the online survey and the clinical assessment were compared regarding sociodemographics, ADHD diagnosis, treatment, and health care utilisation (Table 2). Children of online survey responders were slightly but significantly younger (*p* < 0.001), more likely to live in districts with low or medium socioeconomic deprivation (*p* < 0.001), and less likely to live in districts with high deprivation (*p* < 0.001) compared to non-responders. There were no significant differences with respect to gender (*p* = 0.961). The regional ratios of paediatricians, as well as of medical or psychological psychotherapists and child and adolescent psychiatrists, were slightly but significantly lower at the district level (*p* = 0.001 and *p* = 0.003, respectively). The proportion of participants living in western Germany was higher among responders compared to non-responders (*p* < 0.001). Children of responders were less likely to be incident ADHD cases in 2020 (*p* = 0.002), and among the incident cases, the utilisation of general practitioners was less frequent among them (*p* < 0.001). They were also more likely to have received a prescription of ADHD medication (N06BA04, N06BA09, N06BA02, N06BA12, N06BA21) or behavioural therapy, and showed higher utilisation rates for child and adolescent psychiatrists, psychotherapists, or psychological psychotherapists than children of non-responders (all *p* < 0.001). Generally, albeit significant, the differences between responders and non-responders were small in size. Notable differences were observed only for ADHD medication use and the utilisation of child and adolescent psychiatric or psychotherapeutic services, indicating that parents of children and adolescents with more severe conditions were more likely to participate in the study.

 Numbers not summing up to total due to missing data

**Table 2:** Non-responder analysis (on the basis of administrative data)

	Online sample					Clinical sample				
	Participants^a^ (*n* = 5,461)		Non-participants (*n *= 19,419)			Participants (*n* = 202)		Non-participants (*n* = 24,678)		
	*n* (%)	Mean (SD)	*n* (%)	Mean (SD)	*p*	*n (%)*	Mean (SD)	*n* (%)	Mean (SD)	*p*
**Sociodemographics**										
Age (in years)	-	11.2 (3.2)	-	11.4 (3.5)	< 0.001	-	10.9 (3.1)	-	11.4 (3.4)	0.011
Boys	4,075 (74.6)	-	14,499 (74.7)	-	0.961	145 (71.8)	-	18,429 (74.7)	-	0.389
Deprivation: low	774 (14.2)	-	2,497 (12.9)	-	< 0.001	26 (12.9)	-	3,245 (13.2)	-	0.919
Deprivation: medium	3,659 (67.0)	-	12,613 (65.0)	-	< 0.001	136 (67.3)	-	16,136 (65.4)	-	0.919
Deprivation: high	983 (18.0)	-	4,166 (21.5)	-	< 0.001	39 (19.3)	-	5,110 (20.7)	-	0.919
Federal state: Western Germany	4,695 (86.0)	-	16,266 (83.8)	-	< 0.001	173 (85.6)	-	20,788 (84.2)	-	0.817
Federal state: Eastern Germany	721 (13.2)	-	3,010 (15.5)	-	< 0.001	28 (13.9)	-	3,703 (15.0)	-	0.817
Regional ratio of paediatricians	-	9.4 (3.4)	-	9.5 (3.5)	0.004	-	9.1 (3.2)	-	9.5 (3.5)	0.074
Regional ratio of medical or psychological psychotherapists or child and adolescent psychiatrists	-	6.8 (5.6)	-	7.1 (5.7)	0.003	-	6.9 (5.4)	-	7.0 (5.7)	0.722
**ADHD diagnosis, treatment and health care utilisation**										
ADHD-incident in 2020	1,355 (24.8)	-	5,237 (27.0)	-	0.002	46 (22.8)	-	6,546 (26.5)	-	0.741
Incident ADHD diagnosis: general practitioner	285 (5.2)	-	1,317 (6.8)	-	< 0.001	8 (4.0)	-	1,594 (6.5)	-	0.195
Incident ADHD diagnosis: paediatrician	988 (18.1)	-	3,720 (19.2)	-	0.079	32 (15.8)	-	4,676 (19.0)	-	0.302
Incident ADHD diagnosis: child and adolescent psychiatrist or psychotherapist or psychological psychotherapist	624 (11.4)	-	2,078 (10.7)	-	0.134	19 (9.4)	-	2,683 (10.9)	--	0.580
Incident ADHD diagnosis: other	705 (12.9)	-	2,402 (12.4)	-	0.296	27 (13.4)	-	3,080 (12.5)		0.785
ADHD diagnosis: inpatient	335 (6.1)	-	1,246 (6.4)	-	0.470	9 (4.5)	-	1,572 (6.4)	-	0.334
ADHD medication	3,001 (55.0)	-	8,489 (43.7)	-	< 0.001	84 (41.6)	-	11,406 (46.2)	-	0.213
Behavioural therapy	1,620 (29.7)	-	4,952 (25.5)	-	< 0.001	60 (29.7)	-	6,512 (26.4)	-	0.325
Utilisation of child and adolescent psychiatrist or psychotherapist or psychological psychotherapist (yes/no)	3,341 (61.2)	-	10,601 (54.6)	-	< 0.001	112 (55.5)	-	13,830 (56.0)	-	0.921
Utilisation of child and adolescent psychiatrist or psychotherapist or psychological psychotherapist (frequency)	-	3.5 (3.8)	-	2.9 (3.5)	< 0.001	-	3.2 (3.9)	-	3.0 (3.5)	0.465
Number of prescribed medications in 2020	-	4.6 (3.6)	-	4.5 (3.7)	0.207	-	4.7 (3.4)	-	4.5 (3.7)	0.445

Numbers not summing up to total due to missing data

^a^Responders minus excluded subjects due to missing information for ADHD diagnosis, completion time of less than 657 s, more than 50% missing values, answered unsuitable questionnaire or inconsistency

Youths participating in the clinical assessment were slightly younger than non-participants (Table 2). No other differences were observed between participants and non-participants.

### Weighting

To adjust for the deviations identified in the non-responder analysis, population weights were calculated to normalise the online net sample to the gross sample. The population weights are determined by the inverse probability of an individual participating in the study. Individuals with a low probability of participation represent a larger proportion of the population than those with a high probability. To calculate the weighting factor, a logistic regression model was fitted, including variables identified in the non-responder analysis. The online weights were computed using the R packages survey, srvyr, and svrep [57].

A weight for the clinical subsample was calculated only for subjects for whom a clinical best-estimate judgment was possible (*n* = 201, see above). The clinical weight combines the aforementioned population weight with a correction for the disproportionate sampling of the subsample and thus ensures that findings from the clinical subsample can be generalised to the gross sample. It represents the inverse probability of the clinical judgment, multiplied by the population weight of the online survey. This probability was predicted using a logistic regression model. To adjust for the oversampling of the ED- group in the clinical sampling procedure, the model included information on whether parents reported an ADHD diagnosis for their child. Additionally, an interaction term between gender and parental report was included, as the diagnosis was reported more frequently for boys than for girls. This model was then weighted with the online weight to adjust the clinical subsample to the gross sample.

### Sample characteristics

Table 3 presents the sociodemographic characteristics of the online sample and the clinical subsample. The unweighted mean age of children in the online sample was 12.5 years (SD = 3.22), with a weighted mean age of 12.6 years (SE = 0.05), ranging from 2 to 19 years; 74.6% were male (weighted: 74.1%). In the clinical subsample, the average age was 12.1 years (SD = 3.04), with a weighted mean of 12.4 years (SE = 0.22), ranging from 5 to 19 years; 72.1% were male (weighted: 74.1%). The majority of parents had a medium level of education. In the online sample, 6.3% had a migration background (weighted: 6.5%), compared to 5.0% (weighted: 4.8%) in the clinical subsample. No significant differences were observed between the two samples in terms of sociodemographics, both in the raw data and in the weighted data adjusted to the overall sample. This suggests that the clinical subsample is representative of the participants in the online survey.

**Table 3:** Sample characteristics (on the basis of epidemiological and clinical data)

	Online sample			Clinical subsample		
	*n*	%	%_weighted_^a^	*n*	%	%_weighted_^b^
		(95% CI)	(95% CI)		(95% CI)	(95% CI)
Number of participants	5,461			201^c^		
Age group^d^						
0–2 years	3	0.1 (0.0-0.2)	0.1 (0.0-0.2)	0	0.0	0.0
3–6 years	167	3.1 (2.6–3.5)	3.5 (3.0–4.0)	10	5.0 (2.7-9.0)	3.7 (1.9–7.1)
7–10 years	1,351	24.7 (23.6–25.9)	24.3 (23.1–25.4)	50	24.9 (19.4–31.4)	22.7 (17.1–29.3)
11–13 years	1,827	33.4 (32.2–34.7)	31.2 (30.0-32.5)	74	36.8 (30.4–43.7)	35.8 (29.1–43.1)
14–17 years	1,770	32.4 (31.2–33.7)	33.4 (32.2–34.8)	60	29.8 (23.9–36.6)	33.5 (26.7–41.2)
18–19 years	343	6.3 (5.7-7.0)	7.5 (6.8–8.3)	7	3.5 (1.7–7.2)	4.3 (2.0-9.1)
Gender						
Female	1,386	25.4 (24.2–26.6)	25.9 (24.7–27.1)	56	27.9 (22.1–34.5)	25.9 (20.1–32.7)
Male	4,075	74.6 (73.4–75.8)	74.1 (72.9–75.3)	145	72.1 (65.5–77.9)	74.1 (67.3–79.9)
Parental education (CASMIN)						
Low education	560	10.8 (10.0-11.7)	10.4 (9.6–11.3)	14	7.0 (4.2–11.4)	7.5 (4.3–12.6)
Medium education	3,271	63.1 (61.8–64.4)	63.2 (61.9–64.5)	128	63.7 (56.8–70.1)	65.2 (57.8–71.9)
High education	1,355	26.1 (24.9–27.3)	26.4 (25.1–27.6)	59	29.3 (23.4–36.1)	27.3 (21.3–34.4)
Migration background						
Migration background (two-sided)	332	6.3 (5.7-7.0)	6.5 (5.9–7.3)	10	5.0 (2.7-9.0)	4.8 (2.5–8.9)
No migration background	4,948	93.7 (93.0-94.3)	93.5 (92.7–94.1)	191	95.0 (91.0-97.3)	95.2 (91.1–97.5)

Numbers not summing up to total due to missing data

*CASMIN* Comparative Analysis of Social Mobility in Industrial Nations, *CI* Confidence interval

%_weighted_^a^ = percentage weighted for the online survey, %_weighted_^b^ = clinical weight

^c^The clinical weight was only calculated for subjects for whom a clinical best-estimate judgement was possible (*n =* 201 out of *n =* 202). For reasons of comparability, the unweighted percentages were calculated on the basis of 201 persons

^d^age group at the time of the online survey

## Discussion

The aim of this paper was to describe the study design and conduct, the range of topics covered, the instruments used, and sampling of the project INTEGRATE-ADHD as well as to evaluate the representativeness of the data, to present results on key findings from the non-responder analyses for both the online survey and the online clinical assessment, and to provide an initial sociodemographic description of the sample.

Non-responder analyses were performed in order to assess the differences between those selected for the study and those who ultimately participated. The non-responder analysis for the online survey revealed significant differences with respect to sociodemographics, ADHD diagnosis and treatment, and health care utilisation. Children of responders were slightly younger and less likely to live in socioeconomically deprived areas compared with children of non-responders. This finding is consistent with previous research in child and adolescent surveys, which shows that the parental willingness to participate decreases with increasing age of the children or adolescents and lower socioeconomic status of their families [58, 59].

However, the differences between responders and non-responders were mostly small in magnitude, and the observed statistical significance was largely due to the sample size. Differences between responders and non-responders were more pronounced for ADHD medication use and mental health care utilisation, suggesting that families of children and adolescents with greater symptom severity were more likely to participate.

In contrast, the non-responder analysis for the clinical subsample showed a significant difference only in age, with children and adolescents in the clinical sample being slightly younger than those who did not participate. An in-depth analysis, however, revealed that the proportion of those who had already had an ADHD diagnosis in the previous year was significantly higher among those who consented, were selected, and participated in the clinical assessment. Conversely, the proportion was significantly lower among those whose initial diagnosis was made by a paediatrician (instead of a child psychiatrist or psychotherapist) compared with individuals who had consented and were preselected but did not participate in the clinical assessment for various reasons. It is plausible that an existing patient history increases the willingness to participate in a clinical assessment, as patients are already familiar with the diagnostic process and may encounter fewer barriers or anxieties. Assuming this hypothesis holds true, it is also plausible that families whose children received their initial diagnosis from a paediatrician are less likely to participate. This may be due to the fact that paediatric practices—which often also provide general primary care—may conduct guideline-based diagnostics less likely because of time constraints, compared with child and adolescent psychiatric practices [60].

DAK-Gesundheit is the third-largest statutory health insurance company in Germany, with an insured population that closely approximates the national population. In terms of the representativeness of the INTEGRATE gross sample, a specific comparison with data from all children and adolescents in Germany who are statutorily insured and have an administratively recorded ADHD diagnosis revealed only minor deviations in gender and somewhat larger deviations in age, with younger children overrepresented and older children underrepresented in the INTEGRATE gross sample. However, by design, children of privately insured parents were not included in the study. Therefore, the sample can only be considered approximately representative of the population of statutorily insured children and adolescents with an administrative ADHD diagnosis.

### Strengths and limitations

INTEGRATE-ADHD is the first study in Germany to link administrative, epidemiological, and clinical ADHD diagnostic data for children and adolescents at the individual level. Another strength is the large sample size and its approximate representativeness of children and adolescents with an administrative ADHD diagnosis in Germany.

However, the study has several limitations. Its cross-sectional design does not allow for causal inferences. Potential biases include response bias, for example toward motivated or highly educated parents or those without a migration background; selection bias, such as the potential underrepresentation of severely affected or disadvantaged cases; and self-report bias in parent-reported outcomes. However, non-responder analyses indicated higher participation among parents of more severely affected children and adolescents. As clinical assessments were conducted online only, results may differ from in-person evaluations. In addition, the study uses data from a single statutory insurance company and excludes privately insured children and adolescents, which limits generalisability. The DAK-Gesundheit is the third-largest nationwide statutory health insurance provider in Germany, with an insured population that can be considered approximately broadly representative of German children and adolescent population. To adjust for the deviations identified in the non-responder analyses, weighting factors were calculated. However, the applied weighting cannot fully account for specific ‘not missing at random’ non-response. For example, some parents may have opted not to participate because they were convinced that their child does or does not have ADHD.

## Conclusion

The consortium project INTEGRATE-ADHD represents an important initial step toward integrating multi-source ADHD diagnostic data for children and adolescents in Germany. The project also provides the opportunity to examine health care use, treatment satisfaction, and direct costs associated with an ADHD diagnosis. The results will contribute to a better understanding and evaluation of ADHD diagnostic data from multiple sources, including administrative records, parental or self-reports, and clinical assessments. Furthermore, the project will provide recommendations to improve the quality of diagnosis and treatment for children and adolescents with ADHD, as well as support for their families. Analyses of health care utilisation and associated costs will help identify sustainable, needs-based treatment approaches and reduce the risk of inappropriate care for children and adolescents with ADHD.

While the present study focused on children and adolescents with an administrative ADHD diagnosis, future research could expand on these findings by including children without such records to better capture the full spectrum of ADHD in the population. A follow-up linkage with routine health care and administrative data could help validate reported diagnoses and examine long-term outcomes, but currently still faces legal and data protection challenges. In addition, machine-learning–based approaches could be applied to identify patterns in diagnostic accuracy and treatment trajectories. Finally, longitudinal follow-up of participants would provide insights into the persistence, progression, and outcomes of ADHD over time, thereby enhancing the generalisability and utility of the study findings.

## Data Availability

Access to the datasets generated and analysed during the current study is restricted. The administrative data cannot be made publicly available and its use is limited in duration.

## Abbrevations

ADHD: Attention-deficit/hyperactivity disorder
ASRS: ADHD Self-Report Scale
BELLA: Mental health module of KiGGS
CASMIN: Comparative Analysis of Social Mobility in Industrial Nations
CBCL: Child Behavior Checklist
CES-DC: Centre for Epidemiological Studies Depression Scale for Children
CGI-S: Clinical Global Impressions Scale
CPT: Continuous Performance Test
DADYS-Screen: Diagnostic Tool for Affective Dysregulation in Children—Screening questionnaire
DISYPS: Diagnostic System for Mental Disorders in Childhood and Adolescence
ED+: Youths with epidemiologically parent-reported ADHD diagnosis
ED-: Youths without epidemiologically parent-reported ADHD diagnosis
FBB-ADHS: “Fremdbeurteilungsbogen für Aufmerksamkeitsdefizit-/Hyperaktivitätsstörungen”
GISD: German Index of Deprivation
ICD: International Statistical Classification of Diseases and Related Health Problems
ID: Identification number
ILF(-EXTERNAL): “Interviewleitfaden (für Externale Störungen)”
KiGGS: German Health Interview and Examination Survey for Children and Adolescents
Kinder-DIPS: Diagnostic Interview for Psychiatric Disorders in Children and Adolescents
MAS: “Multiaxiales Klassifikationsschema”
PIN: Personal identification number
PROMIS: Patient Reported Outcomes Measurement Information System
SBB-ADHS: “Selbstbeurteilungsbogen für Aufmerksamkeitsdefizit-/Hyperaktivitätsstörungen”
SCARED: Screen for Child Anxiety-Related Emotional Disorders
SDQ: Strengths and Difficulties Questionnaire
SCL-K-9: Short version of the Symptom Checklist
Zi: Central Research Institute for Ambulatory Health Care in Germany
